# Development of membranoproliferative glomerulonephritis-like glomerulopathy in a patient with neutrophilia resulting from endogenous granulocyte-colony stimulating factor overproduction: a case report

**DOI:** 10.1186/s12882-018-1049-4

**Published:** 2018-10-04

**Authors:** Seigo Ito, Takahiro Uchida, Naoki Oshima, Takashi Oda, Hiroo Kumagai

**Affiliations:** 1 Department of Nephrology and Endocrinology, National Defense Medical College, 3-2 Namiki, Tokorozawa, Saitama 359-8513 Japan; 2 Department of Nephrology, Tokyo Medical University Hachioji Medical Center, Hachioji, Tokyo Japan

**Keywords:** Glomerular endothelial cells injury, Granulocyte-colony stimulating factor (G-CSF), Membranoproliferative glomerulonephritis (MPGN), Neutrophilia

## Abstract

**Background:**

The pathophysiologic role of exogenous granulocyte-colony stimulating factor (G-CSF) administration is reportedly linked to the progression of glomerulonephritis. However, the relationship between endogenous G-CSF overproduction and the progression of glomerulopathy has not been well investigated.

**Case presentation:**

A 76-year-old woman presented with neutrophilia at a medical check-up and thorough examination revealed a high level of serum G-CSF. She subsequently developed mild renal dysfunction and proteinuria. Her renal biopsy showed lobulation of the glomeruli with mesangial proliferation and glomerular capillary walls with a double contour but no immune complex deposition, suggesting membranoproliferative glomerulonephritis-like glomerulopathy. Thereafter, her proteinuria levels fluctuated in parallel with the changes in her blood neutrophil count and finally reduced considerably in association with her decreased neutrophil count.

**Conclusions:**

The unique features of this case suggest that endogenous overproduction of G-CSF could play an important role in the pathogenesis of active glomerulonephritis.

## Background

Recombinant human granulocyte-colony stimulating factor (G-CSF) induces a selective and transient increase in circulating neutrophils by stimulating bone marrow production. It is widely used in patients with neutropenia or to collect progenitor cells for hematopoietic stem cell transplantation [1], and is regarded as a well-tolerated treatment [2].

On the other hand, some adverse effects due to G-CSF administration have been reported, and glomerulonephritis is considered one of them [3–6]. In addition, G-CSF administration has recently been reported to aggravate preexisting glomerulonephritis [7]. However, the relationship between endogenous G-CSF overproduction and the progression of glomerulopathy has not been well investigated.

Herein, we describe a patient with neutrophilia caused by endogenous G-CSF overproduction whose renal biopsy showed membranoproliferative glomerulonephritis (MPGN)-like glomerulopathy. In this case, renal dysfunction and proteinuria developed after the appearance of neutrophilia. The levels of proteinuria fluctuated in parallel with changes in the blood neutrophil count and finally reduced considerably accompanied by decrease in the neutrophil count.

## Case presentation

A 76-year-old Japanese woman presented with neutrophilia, mild renal dysfunction, and proteinuria and was referred to our Department in year X (Table 1). Her neutrophilia was first discovered during a medical check-up when she was 74 years old (year X-2), and thereafter, her neutrophil count progressively increased. Her serum G-CSF was 161 pg/mL (Table 1), which was far beyond the normal range (< 39.0 pg/mL).

**Table 1:** Clinical course in a 76-year-old woman with neutrophilia and proteinuria

Years	X-2	X-1	X	X + 1	X + 4
G-CSF (pg/mL)		161	246		
WBC count (/μL)	13,600	24,700	52,300	42,800	18,600
Neutrophil count (/μL)		20,452	46,861	37,536	13,578
Proteinuria (g/gCr)	< 0.2	< 0.2	2.55	1.57	1.35

*G-CSF* granulocyte-colony stimulating factor, *WBC* white blood cell

Her ^18^F-fluorodeoxyglucose positron emission tomography/computed tomography (^18^F-FDG-PET/CT) scan showed intense uptake in the bone marrow but did not show any evidence of a malignant solid tumor or an occult abscess related to bacterial infection. Fluorescence in situ hybridization was performed on peripheral blood smears, and there was no clonality in the patient’s neutrophils. Her urine had tested positive for urinary occult blood since she was 50 years old, and she developed proteinuria after her neutrophil count increased above 20.0 × 10^3^/μL in year X-1.

Her blood pressure was 118/59 mmHg; other vital signs were also normal. Physical examinations were unremarkable. When she was admitted in year X, the results of laboratory tests were as follows: white blood cell (WBC) count, 35.9 × 10^3^/μL (neutrophil count, 31.3 × 10^3^/μL; 87.2% of the WBC count); hemoglobin level, 11.5 g/dL; platelet count, 27.3 × 10^4^/μL; serum creatinine, 0.85 mg/dL; estimated glomerular filtration rate, 49.4 mL/min/1.73 m^2^; blood urea nitrogen, 16 mg/dL; lactate dehydrogenase, 170 U/L; total protein/albumin, 8.0/3.6 g/dL; and immunoglobulin G (IgG), 2901 mg/dL. Serum immunoelectrophoresis revealed a monoclonal IgG λ peak; however, the levels of other immunoglobulins were normal, and bone marrow aspiration showed less than 10% clonal plasma cells, thereby yielding the diagnosis of monoclonal gammopathy of undetermined significance (MGUS). Tumor markers including carcinoembryonic antigen, squamous cell carcinoma antigen, and cytokeratin fragment were all within normal range. Both the levels of C-reactive protein and complements were normal, and both antinuclear antibody and rheumatoid factor were negative. Tests for hepatitis B virus and hepatitis C virus were negative. Her urinary protein excretion was 2.55 g per gram urinary creatinine, and the proteinuria selectivity index was 0.17. We found hematuria (20–29 red blood cells/high-power field) and granular casts in her urinary sediment.

**Fig. 1:**
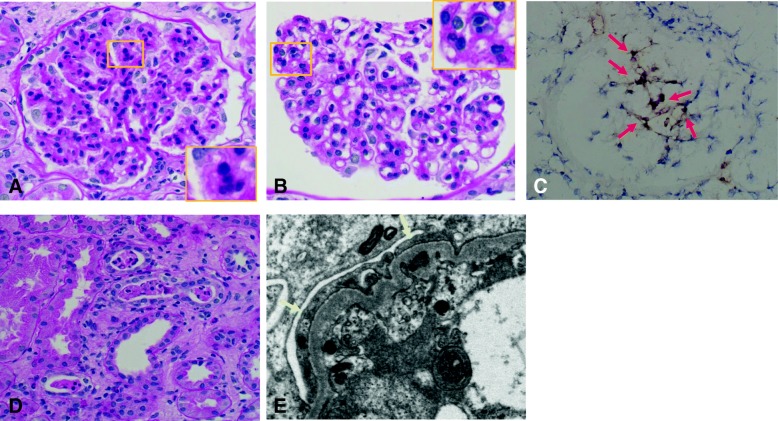
Histological features of the renal biopsy. **a** Lobulation of a glomerulus and mesangial proliferation in light microscopy sections (Periodic acid-Schiff (PAS) stain). Additionally, focal double contouring of the glomerular capillary walls is present (inset). **b** Endocapillary proliferation in another glomerulus (inset). Focal double contouring of the glomerular capillary walls is also observed (PAS stain). **c** Indirect immunoperoxidase staining for neutrophil elastase (NE, DAKO, Santa Clara, CA, US) using fresh-frozen sections clearly shows the infiltration of neutrophils (red arrows). **d** Intratubular cellular casts and expansion of the interstitium (PAS stain). **e** Expansion of the subendothelial space (yellow asterisk) and effacement of podocyte foot processes (yellow arrows) are shown in electron microscopy sections. No electron dense deposits are present (Original magnification, A, B, C, D, 200×)

A renal biopsy was performed, and sections evaluated by light microscopy showed many lobulated glomeruli. Mesangial proliferation and focal double contouring of the glomerular capillary walls were also present (Fig. 1a). Additionally, endocapillary proliferation was observed in the glomeruli (Figs. 1b). Immunoperoxidase staining for neutrophil elastase revealed some neutrophils infiltrating the glomeruli (Fig. 1c). Double immunofluorescence staining for neutrophils and macrophages showed that the infiltration of macrophages was mainly around the glomeruli, and the glomeruli contained more neutrophils than macrophages (data not shown). We identified intratubular cellular casts and interstitial expansion in the tubulointerstitial area (Fig. 1d). Immunoperoxidase staining for G-CSF did not show a specific staining pattern (data not shown). Immunofluorescent staining showed no deposition of immunoglobulins or complements (data not shown). Expansion of the subendothelial space suggesting injury of glomerular endothelial cells and effacement of the podocyte foot processes were seen in sections evaluated by electron microscopy (Fig. 1e). There were no electron dense deposits. Based on these biopsy findings, we diagnosed the patient with MPGN-like glomerulopathy.

The patient was conservatively treated with an angiotensin II type 1 receptor blocker, but her proteinuria did not resolve. On the contrary, her proteinuria fluctuated in accord with the changes in her blood neutrophil count. Finally, the neutrophil count decreased without any medication, and the proteinuria diminished considerably (Table 1).

## Discussion and conclusions

To our knowledge, this is the first reported case of biopsy-proven active glomerulonephritis accompanied by endogenous G-CSF overproduction. The unique feature of this case, that is, the appearance of renal dysfunction and proteinuria after the identification of neutrophilia with G-CSF overproduction, suggested a relationship between the patient’s neutrophilia with G-CSF overproduction and the glomerulopathy. Her clinical course, in which the proteinuria fluctuated in association with changes in the blood neutrophil count, supported this interpretation. As the patient had a history of occult hematuria, it is possible that undiagnosed chronic glomerulonephritis proceeded to active glomerulonephritis through G-CSF overproduction. Indeed, G-CSF administration has recently been shown to aggravate preexisting glomerulonephritis [7].

G-CSF activates neutrophils by increasing CD11b/CD18 expression and elastase activity [7] and enhances their capacity to bind to the endothelium by upregulating E-selectin ligand expression [8]. G-CSF is also known to increase the peripheral blood CD34 count [9] and enhance myeloid cell delivery to inflammatory sites [8]. There have also been reported cases in which membranoproliferative [3], mesangial proliferative [4], and crescentic glomerulonephritis [5] developed after treatment with G-CSF. In mice with experimental glomerulonephritis, G-CSF-deficiency was reported to be renoprotective [10]. Therefore, we hypothesize that G-CSF induced a glomerulopathy that lacked electron-dense immune deposits in this patient. The number of neutrophils or their function might also have impacted the development of the glomerulopathy. Additionally, G-CSF may have indirectly mediated pathogenic kidney processes in this case; previous reports indicate that G-CSF administration exacerbates proteinuria by elevating the level of circulating soluble urokinase plasminogen activator receptors [11, 12]. On the other hand, G-CSF has been reported to exert renoprotective effects in animal models of diabetic nephropathy [13]. A difference in species or disease models might account for this discrepancy. Additionally, the possibility that G-CSF overproduction and glomerulopathy were coincidental findings in our patient is undeniable. Further experimental research to investigate this matter is needed.

G-CSF-producing tumors have been reported in various types of cancer including lung, urinary tract, and digestive tract [14]. However, our patient’s measured tumor markers were all within the normal range, and the ^18^F-FDG-PET/CT scan did not show any malignant solid tumors. Intense uptake in her bone marrow was shown by ^18^F-FDG-PET/CT, but this finding could be explained by the high bone marrow metabolism associated with excessive granulocyte production [15]. Additionally, hematopoietic malignancy was ruled out by bone marrow aspiration, and blood neutrophil count of the present case rather decreased without any medication. We, therefore, could not specify the sites of G-CSF overproduction in our patient. However, it should be taken into consideration that a malignancy may emerge in the future. Although immunoperoxidase staining for G-CSF did not show a specific staining pattern, the possibility of local G-CSF production in the kidneys should also be kept in mind. We did not evaluate the renal G-CSF mRNA level in our patient. However, it was reported that murine mesangial cells could express G-CSF mRNA [16].

Saigusa et al. previously reported a case of essential thrombocythemia associated with severe renal glomerular endothelial cell injury and glomerulosclerosis with mesangial proliferation [17]. The concept of myeloproliferative neoplasm (MPN)-related glomerulopathy, characterized by mesangial proliferation, features of chronic thrombotic microangiopathy (TMA) including focal double contouring of the glomerular capillary walls, and intracapillary hematopoietic cell infiltration, was subsequently proposed [18]. Indeed, although our patient did not have neutrophil monoclonality, the pathological features of her case mimic the characteristics of MPN-related glomerulopathy.

The present patient was diagnosed with MGUS. Furthermore, it has been recently reported that in elderly patients, monoclonal immunoglobulinemia is closely associated with C3 glomerulopathy (C3G) or atypical hemolytic uremic syndrome (aHUS) [19]. Indeed, both C3G and aHUS are the differential diagnoses of the MPGN pattern of injury. However, both C3G and aHUS were unlikely in our patient for the following reasons: firstly, no deposition of complements was shown by immunofluorescent staining, and there were no electron dense deposits, as determined by electron microscopy. Secondly, the clinical features of TMA were absent. Finally, the levels of complements were normal.

Lowering the G-CSF level and neutrophil count might have been an effective therapeutic approach in the present case. However, effective therapies for such purpose have not been established, especially in cases with G-CSF overproduction of unknown origin. It has been reported that steroids may not be effective when treating MPN-related glomerulopathy [18]. Further studies will be required in the future to develop an effective therapy.

In conclusion, we report a case of neutrophilia caused by endogenous G-CSF overproduction complicated by the development of MPGN-like glomerulopathy. Our findings suggest that endogenous overproduction of G-CSF could play an important role in the pathogenesis of active glomerulonephritis.

## Abbreviations

^18^F-FDG-PET/CT: ^18^F-fluorodeoxyglucose positron emission tomography/computed tomography
G-CSF: Granulocyte-colony stimulating factor
IgG: Immunoglobulin G
MPGN: Membranoproliferative glomerulonephritis
MPN: Myeloproliferative neoplasm
WBC: White blood cell

## References

[1] Deotare U, Al-Dawsari G, Couban S, Lipton JH (2015). G-CSF-primed bone marrow as a source of stem cells for allografting: revisiting the concept. Bone Marrow Transplant.

[2] Peddi VR, Hariharan S, Schroeder TJ, First MR (1996). Role of granulocyte colony stimulating factor (G-CSF) in reversing neutropenia in renal allograft recipients. Clin Transpl.

[3] Magen D, Mandel H, Berant M, Ben-Izhak O, Zelikovic I (2002). MPGN type I induced by granulocyte colony stimulating factor. Pediatr Nephrol.

[4] Bonilla MA, Dale D, Zeidler C, Last L, Reiter A, Ruggeiro M (1994). Long-term safety of treatment with recombinant human granulocyte colony-stimulating factor (r-metHuG-CSF) in patients with severe congenital neutropenias. Br J Haematol.

[5] Sotomatsu M, Kanazawa T, Ogawa C, Watanabe T, Morikawa A (2000). Complication of rapidly progressive glomerulonephritis in severe congenital neutropenia treated with long-term granulocyte colony-stimulating factor (filgrastim). Br J Haematol.

[6] Nasilowska-Adamska B, Perkowska-Ptasinska A, Tomaszewska A, Serwacka A, Marianska B (2010). Acute glomerulonephritis in a donor as a side effect of allogeneic peripheral blood stem cell mobilization with granulocyte colony-stimulating factor. Int J Hematol.

[7] Batal I, Markowitz GS, Wong W, Avasare R, Mapara MY, Appel GB (2016). Filgrastim-induced crescentic transformation of recurrent IgG2λ GN. J Am Soc Nephrol.

[8] Dagia NM, Gadhoum SZ, Knoblauch CA, Spencer JA, Zamiri P, Lin CP (2006). G-CSF induces E-selectin ligand expression on human myeloid cells. Nat Med.

[9] Sloand EM (2005). Soluble urokinase activator receptor (suPAR) in stem cell mobilization. Blood.

[10] Kitching AR, Ru Huang X, Turner AL, Tipping PG, Dunn AR, Holdsworth SR (2002). The requirement for granulocyte-macrophage colony-stimulating factor and granulocyte colony-stimulating factor in leukocyte-mediated immune glomerular injury. J Am Soc Nephrol.

[11] Gallon L, disease QSEG (2017). A suPAR kidney connection found in the bone marrow. Nat Rev Nephrol.

[12] Hahm E, Wei C, Fernandez I, Li J, Tardi NJ, Tracy M (2017). Bone marrow-derived immature myeloid cells are a main source of circulating suPAR contributing to proteinuric kidney disease. Nat Med.

[13] Erbas O, Yapislar H, Oltulu F, Yavasoğlu A, Aktug H, Taskiran D (2014). Nephro-protective effect of granulocyte colony-stimulating factor in streptozotocin induced diabetic rats. Biotech Histochem.

[14] Yasui H, Sato K, Takeyama Y, Ando A, Kato T, Hashimoto H (2014). Granulocyte colony-stimulating factor-producing carcinoma of unknown primary site. Case Rep Oncol.

[15] Morooka M, Kubota K, Murata Y, Shibuya H, Ito K, Mochizuki M (2008). (18)F-FDG-PET/CT findings of granulocyte colony stimulating factor (G-CSF)-producing lung tumors. Ann Nucl Med.

[16] Jadus MR, Pai R, Horansky E, Wepsic HT, Kirschenbaum MA, Kamanna VS (1994). Hematopoietic colony stimulatory factor formation by murine mesangial cells: gene expression and biological activity. Biochim Biophys Acta.

[17] Saigusa T, Kikuchi Y, Yamada M, Imakiire T, Hyodo T, Suzuki S (2006). A case of essential thrombocytosis developing nephrotic syndrome and severe endothelial damage. J Nephrol.

[18] Said SM, Leung N, Sethi S, Cornell LD, Fidler ME, Grande JP (2011). Myeloproliferative neoplasms cause glomerulopathy. Kidney Int.

[19] Sethi S, Rajkumar SV, D'Agati VD (2018). The complexity and heterogeneity of monoclonal immunoglobulin-associated renal diseases. J Am Soc Nephrol.

